# Heart sound classification based on improved mel-frequency spectral coefficients and deep residual learning

**DOI:** 10.3389/fphys.2022.1084420

**Published:** 2022-12-22

**Authors:** Feng Li, Zheng Zhang, Lingling Wang, Wei Liu

**Affiliations:** ^1^ Department of Computer Science and Technology, Anhui University of Finance and Economics, Bengbu, Anhui, China; ^2^ School of Information Science and Technology, University of Science and Technology of China, Hefei, Anhui, China

**Keywords:** heart sound classification, cardiovascular, MFCC, deep learning, Resnet

## Abstract

Heart sound classification plays a critical role in the early diagnosis of cardiovascular diseases. Although there have been many advances in heart sound classification in the last few years, most of them are still based on conventional segmented features and shallow structure-based classifiers. Therefore, we propose a new heart sound classification method based on improved mel-frequency cepstrum coefficient features and deep residual learning. Firstly, the heart sound signal is preprocessed, and its improved features are computed. Then, these features are used as input features of the neural network. The pathological information in the heart sound signal is further extracted by the deep residual network. Finally, the heart sound signal is classified into different categories according to the features learned by the neural network. This paper presents comprehensive analyses of different network parameters and network connection strategies. The proposed method achieves an accuracy of 94.43% on the dataset in this paper.

## 1 Introduction

Cardiovascular disease is a term used to describe a group of diseases, including coronary heart disease, cerebrovascular disease, and rheumatic heart disease. A patient’s blood pressure, blood sugar, and lipid levels can be raised by fried foods, fast foods, alcohol, and tobacco, as well as weight gain and obesity, leading to premature death. Prevention of sudden death from cardiovascular disease can be achieved by finding groups at risk for cardiovascular disease and ensuring they receive the proper treatment. It is possible to reduce the risk of sudden death from cardiovascular disease by reducing alcohol consumption, reducing salt intake, eating more fruits and vegetables, and exercising more.

**FIGURE 1:**
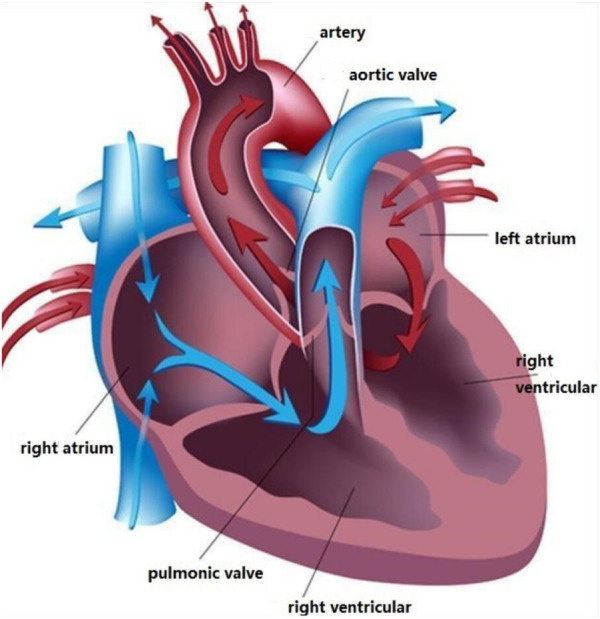
The Structure of the human heart.

Heart sounds are produced by the heart through rhythmic contraction and diastole. The heart is the powerhouse of the body and it is the most critical organ in the body, responsible for delivering blood to other organs to provide oxygen and other nutrients and to carry away the end products of metabolism so that cells can maintain a normal physiological state. Hearts have four chambers: Left atrium, left ventricle, right atrium, and right ventricle, the details of heart structure are shown in Figure 1. Atrioventricular valves prevent blood from flowing backward between the atria and ventricles Li S. et al. (2020).

**FIGURE 2:**
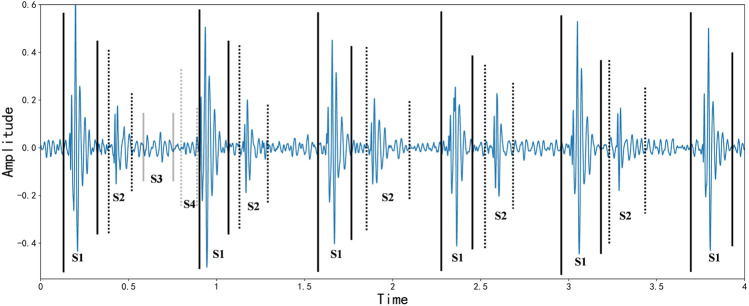
Waveform representation of S1, S2, S3, and S4 sounds in systole and diastole intervals, as of Varghees and Ramachandran (2014).

A cardiac cycle occurs when one heartbeat precedes the next, producing four heart sounds, which are the first, second, third, and fourth heart sounds. Screening for cardiovascular disease by auscultatory heart sound auscultation is a simple, necessary, and effective method that has been used for over 180 years Liu et al. (2016). The first heart sound marks the beginning of ventricular systole and is characterized by long duration, high intensity, and loud sound. The second heart sound marks the beginning of ventricular diastole and has the characteristics of shorter duration, less intensity, and less sound. After the second heart sound, the third heart sound occurs. It lasts between 0.04 and 0.05 s and has a longer wavelength. About half of young adults and most children hear it, and it does not necessarily indicate abnormality. In the fourth heart sound, a long wave sound precedes the first heart sound and lasts for about 0.04 s. It is mechanical wave caused by the contraction of the atria and the rapid filling of the ventricles with blood flow, also known as an atrial sound. Most healthy adults can record a tiny fourth heart sound on an electrocardiogram, which is difficult to detect on general auscultation. Based on the patient’s clinical condition, the physician records the four basic heart sounds and analyzes their differences from the normal situation. It is typically tricky for physicians to determine a patient’s condition by heart sound auscultation in clinical practice Jiang and Choi (2006). Industrialization has made sophisticated machines standard medical tools, and electrocardiograms (PCG) are recorded using acoustic instruments to diagnose and treat patients. With the continuous application of PCG, the use of signal processing and artificial intelligence techniques to extract physiological and pathological information from PCG data has gradually become a popular trend Herzig et al. (2014). Benefit from the development of deep learning field in recent years Hinton and Salakhutdinov (2006); Yu et al. (2013); Ranzato et al. (2006); Bengio. (2009); Hinton and Salakhutdinov (2012); Vincent et al. (2010); Silver et al. (2016); Nair and Hinton (2010), a new horizon has been opened for heart sound classification Zhang and Han (2017). CNN is now a mature deep learning framework since it was first proposed in 2006. It has become a widely used approach in computer vision due to its convolutional layer that learns local patterns of images. CNN is also gradually applied to biomedical signal classification and speech semantic recognition through corresponding audio processing methods, such as transforming human physiological signals into speech spectrograms. Recurrent neural networks (RNN) are a class of neural networks that specialize in processing sequential data. Gated recurrent units (GRU) and long short-term memory (LSTM) are improved versions of RNN, and they provide state-of-the-art performance in many applications, including machine translation, speech recognition, and image captioning Abduh et al. (2019). Heart sound signals are sequential data with strong temporal correlation, so heart sound classification can be efficiently processed by RNN Nogueira et al. (2019); Ismail et al. (2022); Sakib et al. (2019). Figure 2 describes the Waveform representation of S1, S2, S3, and S4 sounds in systole and diastole intervals.

**FIGURE 3:**
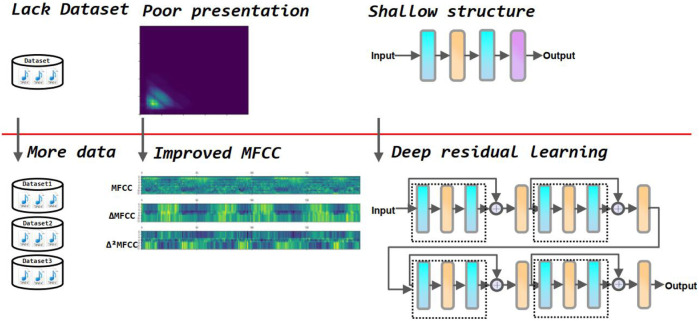
Motivation of the proposed method.

In addition, since some noise in the environment is inevitably collected during the acquisition of heart sounds, this can greatly affect the accuracy of the model classification. Therefore, it is crucial to process the original heart sound signal through feature engineering before feeding it into the neural network for training. There are several commonly used feature extraction methods in heart sound classification tasks, including discrete wavelet transform coefficients (DWT) Mei et al. (2021), and Mel frequency cepstral coefficients (MFCC) Yang and Hsieh (2016). In this paper, the MFCC-based first and second-order difference coefficients are used as the input tensor of the neural network. This feature extraction method reduces the effect of noise on the results and allows the neural network to extract the physiological and pathological features in the heart sound signal, resulting in higher classification accuracy. Compared to traditional heart sound classification algorithms, deep learning techniques avoid the problems of manual intervention, complex processes, and poor generalization. Kui et al. (2021) combined MFSC and CNN for classification of heart sounds. Li et al. (2021) used Short Time Fourier Transform (SFTF) based features as input to CNN. Tschannen et al. (2016) used Wavelet-based features and CNN. Li F. et al. (2020) extracted 497 features from time series as input to the CNN. Er (2021) proposed Local Binary Pattern (LBP) and Local Ternary (LTP) pattern features as input to the CNN. Wu et al. (2019) used MFCC as input to the CNN. Lack of large authoritative open heart sound datasets restricts the performance of the model. To address this concern, this paper incorporates three of the most widely used heart sound datasets. It helps to radically improve the performance of the deep learning model. Although the performance of the above methods has been greatly improved compared to traditional machine learning methods, most of these are shallow structures and the features used are insufficient to fully express the information of heart sounds. In this study, we select improved MFCC as input features to more comprehensively represent the static and dynamic characteristics of the heart sound signal. Additionally, we use a residual neural network which alleviates gradient disappearance and degradation during training. Figure 3 summarizes the motivation of our study.

The rest of the paper is structured as follows: Section 2 discusses recent research trends and essential methods related to heart sound classification. Section 3 describes in detail the preprocessing and feature engineering of heart sound audio and introduces the deep residual neural network structure used in this paper and analyzes in detail the more critical convolution and residual principles. In Section 4, we describe the three datasets used in this paper in detail. We split 20% of the dataset as the testing set. All metrics are the results of the testing set. Additionally, we make a comparison between MFCC, ▵MFCC, ▵^2^MFCC, and improved MFCC to further explain what the improvements are for a better understanding of the superiority of the methods in this paper, RNNs and CNNs are used for comparison and we show models’ loss and accuracy during training. We also list references with other methods used for comparison. Section 5 summarizes our study, and our proposed method is feasible for the heart sound classification task.

## 2 Related work

At present, heart sound auscultation technology is one of the leading clinical diagnostic tools for treating cardiovascular diseases, with the characteristics of non-invasive, efficient, convenient, and can obtain physiological and pathological information about the heart, but due to the complex clinical diagnostic conditions, there is a lot of noise pollution, a lack of experience in physicians are often disturbed by the noise of the environment, resulting in an inaccurate diagnoses of the condition. In 1929, the German doctor Werner used a catheter to deliver drugs to the heart, opening the door to the use of physical models to study cardiovascular disease; in the 1970s, Dr. Marcus in the United States used angiography to observe the causes of cardiovascular disease, overturning long-held misconceptions about heart disease; in the 1980s, the earliest cardiac defibrillators came into clinical use at Johns Hopkins University, and the earliest telemetry systems were developed so that Doctors coule observe the vital signs of heart disease patients from a distance; in recent years, with the development of technology, devices similar to comprehensive ECG heart sound analyzers and intelligent electronic stethoscopes have been put into clinical use, but due to the inevitable factors in the use process, the collected heart sound signals will contain various types of noise to varying degrees, affecting the final diagnostic results. At present, digital filters, wavelet decomposition and empirical modal decomposition are widely used for digital denoising of heart sound signals. In recent years, with the rise of artificial intelligence, big data, and other technologies, more accurate and effective heart sound detection methods are expected to be realized.

The dataset is one of the fundamental issues affecting the results, and heart sound classification is no exception. In general, the larger the data set, the more specialized the distribution, and the more extensive the heart sound data, the more overfitting of the model can be avoided, and the generalizability of the model can be increased. According to a survey Milani et al. (2022), using deep learning techniques for heart sound classification tasks remains challenging due to the lack of a large authoritative open heart sound dataset. In this paper, the Physio heart sound dataset Liu et al. (2016), Pascal heart sound dataset Gomes et al. (2013) and Yaseen heart sound dataset Son and Kwon (2018) were used to construct more extensive, less noisy, and more reliable heart sound dataset. Positive and negative sample imbalances can affect the performance of the model. It is assumed that the distribution of positive and negative samples in the feature space is unbalanced. When the neural network tries to learn the mapping relationship model. It predicts that more samples will bring less loss in most feature space regions. Eventually, this causes the model to fail, and the predicted values are always concentrated near the labels with more samples. That is, the model has very high accuracy on the training set, but a low accuracy on the validation and test sets. It significantly reduces the generalizability of the model. To solve such problems, researchers usually sample the heart sound data and perform slicing operations Baghel et al. (2020); Baydoun et al. (2020) to ensure the balance between the different labels of the samples. Wang et al. (2021) used a weighted improvement of the classifier to reduce the impact of the unbalanced dataset on training. In this paper, the pre-processing of heart sound audio is used to perform cuts and enhance a smaller number of samples to avoid the problem of sample imbalance.

In general, binary classification, multiple classification and regression are often used in classification problems, and how the classification task is chosen can also affect the classification results to some extent. For sequence data with considerable background noise such as heart sounds, the impact of the acquisition process on the real heart sounds must be considered according to the actual situation of the data set. In the current studies of heart sound classification, most of the tasks are dichotomous, normal heart sounds and abnormal heart sounds. Few experiments have classified specific situations such as aortic stenosis and mitral valve insufficiency based on medical knowledge. Demir et al. (2019) used deep convolution neural networks to perform a four classification task on a Kaggle dataset, as well as Oh et al. (2020) performed a quintuple classification task on a heart sound dataset. In this paper, heart sound datasets from three different platforms are considered, considering the inevitable noise generated during the acquisition process due to hardware limitations. Since some cannot identify the heart sound signals, three classification tasks are performed for heart sounds, namely normal, abnormal and noisy, and this selection of classification tasks is closer to the actual situation. It also helps to further improve the accuracy and practical application of heart sound classification.

Many researcher have used deep learning techniques to solve heart sound classification problems. Kui et al. (2021) investigated the effect of discrete cosine transform (DCT) on classification results during MFCC signal extraction. MFSC is an intermediate state in the MFCC extraction process, which omits the step of DCT. CNN is essentially a non-linear transformation of the data, and since DCT is essentially a linear transformation, this operation results in the absence of pathological information in the heart sound signal, so MFSC is feasible for heart sound classification using deep learning techniques. Krishnan et al. (2020) obtained an accuracy of 85.74% by directly using the unsegmented PCG signal as the input to the CNN. Zeinali and Niaki (2022) used a heart sound audio signal processing algorithm to convert one-dimensional temporal features into two-dimensional spectral features. This proposed method achieved 87.0% accuracy in a heart sound triple classification task. Tian et al. (2022) directly trained the neural network using raw data without using feature engineering from the PhysioNet dataset to perform a binary classification task on PCG to distinguish between normal and abnormal heart sounds. Wang et al. (2021) extracted five classes of features by segmenting the PCG signal. and used a recursive feature elimination method to obtain suitable input features, and proposed an XGBoost-based and LSTM combination for heart sound classification, and obtained an accuracy of 90.0% on the test set. Li et al. (2021) segmented the original heart sound signal and then calculated its frequency domain features by short-time Fourier transform. For training, they proposed 2D-CNN and achieved an accuracy of 85.70%. Er (2021) extracted the local binary pattern (LBP) of heart sounds using local three-valued pattern (LTP) and trained it with 1D-CNN with an accuracy of 90% on the PhysioNet dataset. Ren et al. (2022) used the attention mechanism to explore the interpretable heart sound classification algorithm for heart sound triple classification task on PhysioNet dataset and obtained an unweighted average recall of 51.2%. Iqtidar et al. (2021) obtained 98.3% accuracy on heart sound double classification problem using MFCC based 1D adaptive local ternary model and support vector machine. Lahmiri and Bekiros (2022) used discrete wavelet transform with support vector machine optimized through bayesian optimization obtained 89.26% accuracy. In the heart-tone classification task mentioned above, neural networks with MFCC-based features perform better. To further enhance the advantages of MFCC features in expressing heart sound signals, this paper calculates first-order and second-order difference coefficients for expressing the dynamic properties of heart sound signals.

## 3 Proposed methodology

**FIGURE 4:**
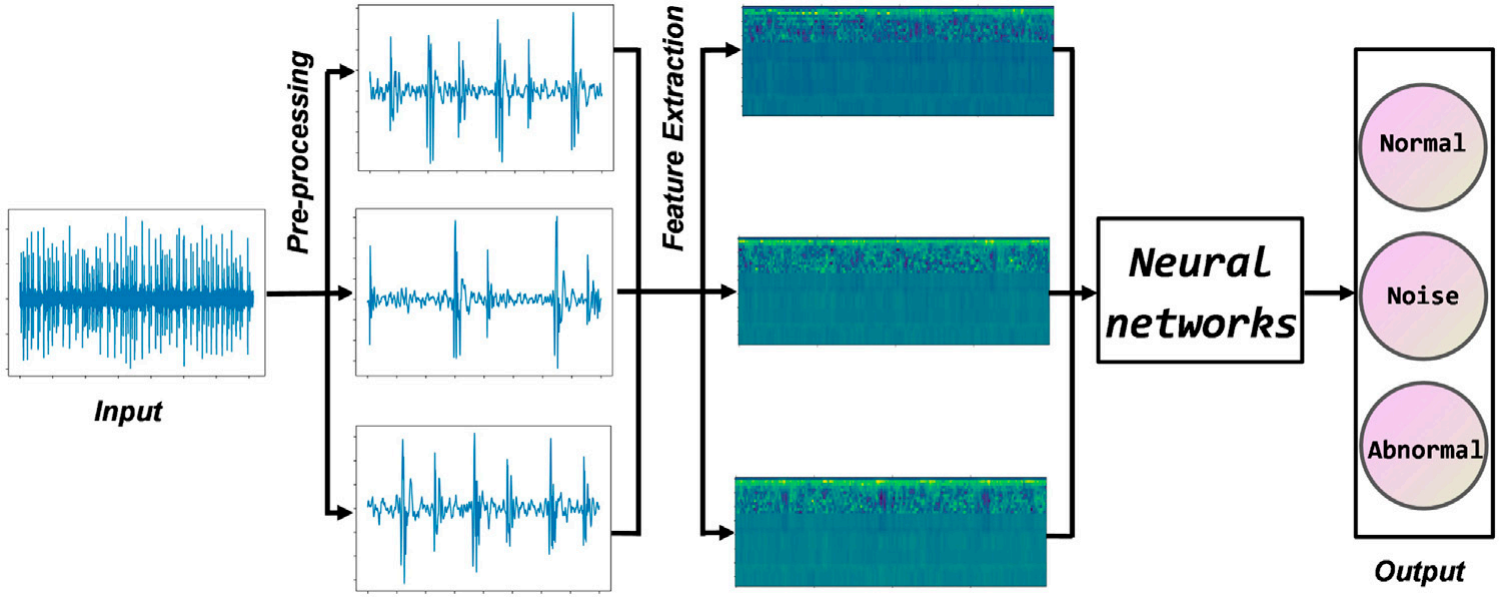
Flow chart of the proposed method.

This section describes the heart sound classification algorithm proposed in this paper in three parts. The first step is data set fusion, which filters, downsamples, and cuts the original heart sounds. The second step is feature engineering, extracting standard MFCC, first-order MFCC, and second-order MFCC, and fusing them into input feature vectors. In the third step, a deep residual neural network is constructed, and feature vectors are input for training. Finally, the test samples are predicted using the trained model, and the accuracy is counted. Figure 4 shows the workflow of this paper. The innovation of the methodology as threefold: 1) Using the authoritative heart sound datasets from three different sources, which helps to radically improve the performance of the deep learning model. 2) Selecting improved MFCC as input features to more comprehensively represent the static and dynamic characteristics of the heart sound signal. 3) Using a residual neural network, which alleviates gradient disappearance and degradation during training.

### 3.1 Dataset fusion

The label classification standards of the datasets selected in this paper are different. Before entering the data into the neural network, the labels must be unified, and data pre-processing is performed on all files. making full use of heart sound datasets from different sources helps to improve the generalization of the model further. According to the characteristics of the label types of the dataset, this paper divides the labels of the fused heart sound data into three categories: normal, abnormal, and noise.

#### 3.1.1 Digital filtering

In collecting heart sound audio, due to hardware limitations and the influence of the background environment, many noises will inevitably be collected in the audio. To reduce the impact of noise on neural network training, this paper filtered the heart sound audio. To preserve the low frequency components of heart sounds that contains important physiological information, this paper sends the heart sound audio into the fifth-order 400 hz Butterworth low-pass filter to filter out the high-frequency murmurs in the heart sound signal.

#### 3.1.2 Down sampling

To reduce the computational complexity of the model and ensure that the heart sound data from different sources can generate the same size feature map in the subsequent feature engineering, all audio signals are down-sampled to 2000 hz.

#### 3.1.3 Audio cutting

Considering the significant difference in length between heart sound audios, this paper cuts the audio in units of 2 s to use the existing heart sound audio and unified audio length as much as possible. On the other hand, considering the solid temporal correlation of pathological features in heart sound audio, heart sound audio with too short duration is difficult to express the pathological features of heart sound, so this paper discarded heart sound audio with less than 2 s.

### 3.2 Feature engineering

In most cases, deep learning models cannot learn from completely arbitrary data, so it is essential to extract heart sound features by hard coding through feature engineering. To obtain an effective pathological feature representation of cardiovascular disease, this paper used an improved feature extraction algorithm based on MFCC Deng et al. (2020). The human ear’s perception of frequency is logarithmic. It is sensitive to changes in low-frequency bands and insensitive to changes in high-frequency bands. The use of linearly distributed spectrograms in feature engineering affected the model’s performance. MFCC reflects the non-linear relationship between the human ear and the sound frequency, which can effectively extract the pathological features in the heart sound audio. The calculation formula of the MFCC is shown as follows

$$Mel\left(f\right)=2595\mathrm{lg}\left(1+f/700\right)$$ (1)

where lg is defined as the base 10 logarithm.

#### 3.2.1 Signal pre-emphasis

In processing the heart sound signals, the high-frequency signal generated during cardiovascular exercise is inadequate, and the low-frequency signal is adequeate. The reason for this phenomenon can explain from the physical level. In the process of sound energy propagation in the medium, the higher the frequency, the more it is easy to be lost, and pre-emphasis makes up for the loss of high frequency and protects the original heart sound signal. In this paper, the heart sound signal. is passed through a high-pass filter to narrow the intensity gap between the high and low-frequency components of the signal. The specific operation of the signal x[n] is shown as follows

$$y\left[n\right]=x\left[n\right]-\alpha x\left[n-1\right]$$ (2)

where *α* usually takes a value close to 1.

#### 3.2.2 Framing windowing

To obtain the distribution of each element of frequency in the heart sound audio, it is necessary to perform Fourier transform on the audio signal, and the Fourier transform requires that the input signal must be stable, so the audio signal needs to be framed and windowed first. Framing is to divide the original signal into several small blocks according to time, and one block is called a frame. In framing process, the original signal will have a spectrum leakage phenomenon. The spectrum corresponding to the original signal and the signal after framing are very different. The Hamming window can effectively overcome the leakage phenomenon Astuti et al. (2012). The Hamming window function W(n) is shown as follows

$$W\left(n\right)=\left(1-\alpha \right)-\alpha \cos\left(2\pi n/\left(N-1\right)\right),0\leq n\leq N-1$$ (3)

where the *α* value is 0.46 by suggested in Trang et al. (2014).

#### 3.2.3 Get power spectrum

After framing and windowing, this paper used discrete Fourier transform (DFT) on the data to transform the time-domain signal into a frequency-domain signal to obtain the spectrum X(k) is shown as follows

$$X\left(k\right)=\overset{N-1}{\underset{n=0}{\sum }}x\left(n\right)e^{-j2\pi nk/N},0\leq n,k\leq N-1$$ (4)

The power spectrum P(k) is equal to the signal spectrum X(k) as the square of its modulus, as shown in Eq. 5. The power spectrum expresses the energy characteristics of the heart sound signal more accurately, retains some amplitude elements in the heart sound spectrum, and discards the phase characteristics of the heart sound spectrum is described as follows

$$P\left(k\right)=\frac{1}{N}|X\left(k\right){|}^{2}$$ (5)

#### 3.2.4 Mel filter bank

A normal human ear is able to hear sounds with frequencies from 20 Hz to 20,000 Hz. The range of 20 Hz to 20,000 Hz is called the audible frequency range. The sounds we hear comprise of various frequencies. The Mel filter bank is represented as a group of triangular filters on the image. Usually a set contains 20 to 40 ascending triangular filters, and the starting position of each triangular filter is at the midpoint of the previous triangular filter, and because it has a linear frequency in the Mel scale, it is called a Mel filter bank. At each frequency, calculate the product of P(k) and filter Hm(k). Defining a triangular filter bank with Mel filters, the frequency response Hm(k) of the triangular filter is calculated as follows

$$H_{m}\left(k\right)=\left\{\begin{array}{cc} 0, & k<f\left(m-1\right)\\ \frac{k-f\left(m-1\right)}{f\left(m\right)-f\left(m-1\right)}, & f\left(m-1\right)\leq k\leq f\left(m\right)\\ \frac{f\left(m+1\right)-k}{f\left(m+1\right)-f\left(m\right)}, & f\left(m\right)\leq k\leq f\left(m+1\right)\\ 0, & k>f\left(m+1\right) \end{array}\right.$$ (6)

where m represents the serial number of the filter, and f (m-1), f(m), and f (m+1) correspond to the starting point, middle point, and end point of the filter, respectively. In calculations, the values of m take 1, 2, … , 13. For a Mel triangular filter, f(m) represents the center frequency of the Mel trangular filter, f (m-1) represents the start of the Mel trangular filter, and f (m+1) represents the end of the Mel trangular filter. Summing the whole of Hm(k), we can obtain Eq. 7, and the value of *M* is 13.

$$\overset{M-1}{\underset{m=0}{\sum }}H_{m}\left(k\right)=1$$ (7)

#### 3.2.5 Log spectrum

The logarithmic energy spectrum S(m) at each frame is obtained by using the logarithmic operation is shown as follows

$$S\left(m\right)=\ln\left[\overset{N-1}{\underset{k=0}{\sum }}P\left(k\right)H_{m}\left(k\right)\right],0\leq m\leq M$$ (8)

where lg is defined as the base *e* logarithm.

#### 3.2.6 Discrete cosine transform

The discrete cosine transform (DCT) is performed on the above log spectrum to obtain the Mel cepstral coefficient C(n), which is the MFCC feature, The corresponding equation is described as follows.

$$C\left(n\right)=\overset{N-1}{\underset{m=0}{\sum }}S\left(m\right)\cos\left(\pi n\left(m-0.5\right)/M\right),\;n=1,2,\ldots ,L$$ (9)

#### 3.2.7 Dynamic feature extraction

**FIGURE 5:**
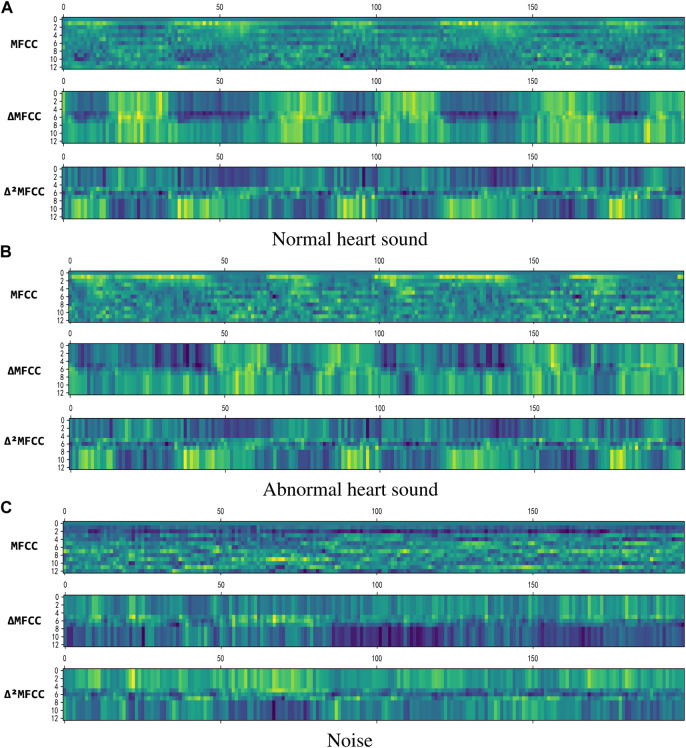
2D visualization of the features. **(A)** Normal heart sound. **(B)** Abnormal heart sound. **(C)** Noise

MFCC reflects the static information of the heart sound signal, and the dynamic information of the heart sound signal also contains rich pathological features, which can be used to improve the classification accuracy further. To reflect the dynamic information of the heart sound signal, this paper extracts the first-order difference coefficient D(n) and the second-order difference coefficient D2(n) based on MFCC. The calculation formulas are described as follows

$$D\left(n\right)=\frac{1}{\sqrt{\sum_{i=-k}^{i=k}i^{2}}}\overset{i=k}{\underset{i=-k}{\sum }}i\cdot C\left(n+i\right)$$ (10)

$$D_{2}\left(n\right)=\frac{1}{\sqrt{2\sum_{i=-k}^{i=k}i^{2}}}\overset{i=k}{\underset{i=-k}{\sum }}i\cdot D\left(n+i\right)$$ (11)

where the value of k is taken as 2, and C (n + i) is a frame of MFCC coefficient. Figure 5 shows 2D visualization of them, where MFCC is the result of Eq. 9, ▵MFCC is the result of Eq. 10, and ▵^2^MFCC is the result of Eq. 11. The size of them are all (199,13), we use them to construct a (199,39) feature as the input of neural network.

### 3.3 Resnet

**FIGURE 6:**
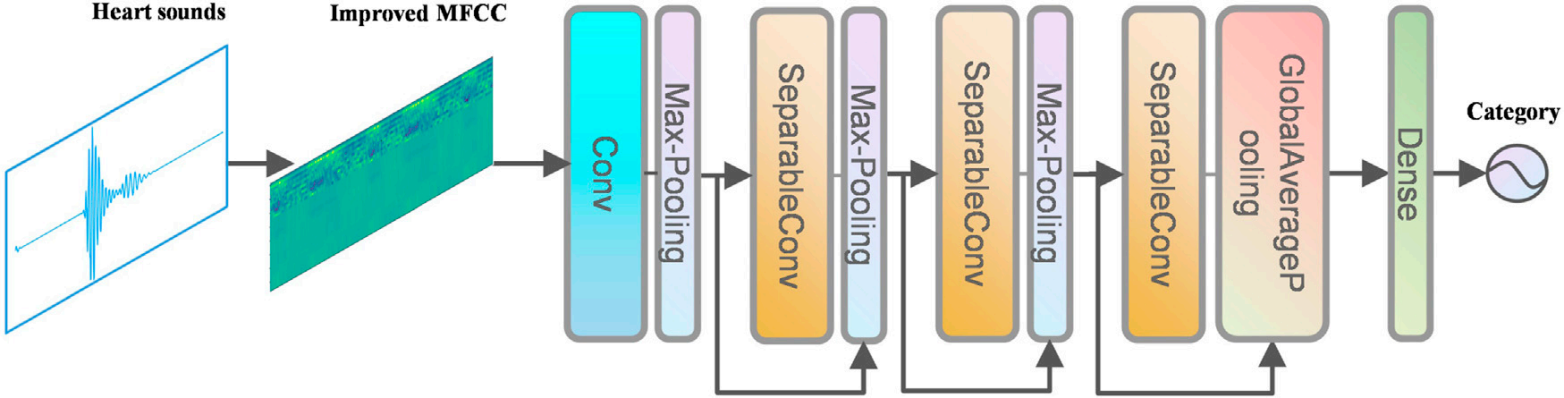
Structure of Resnet.

The network structure in this paper is shown in Figure 6.

**FIGURE 7:**
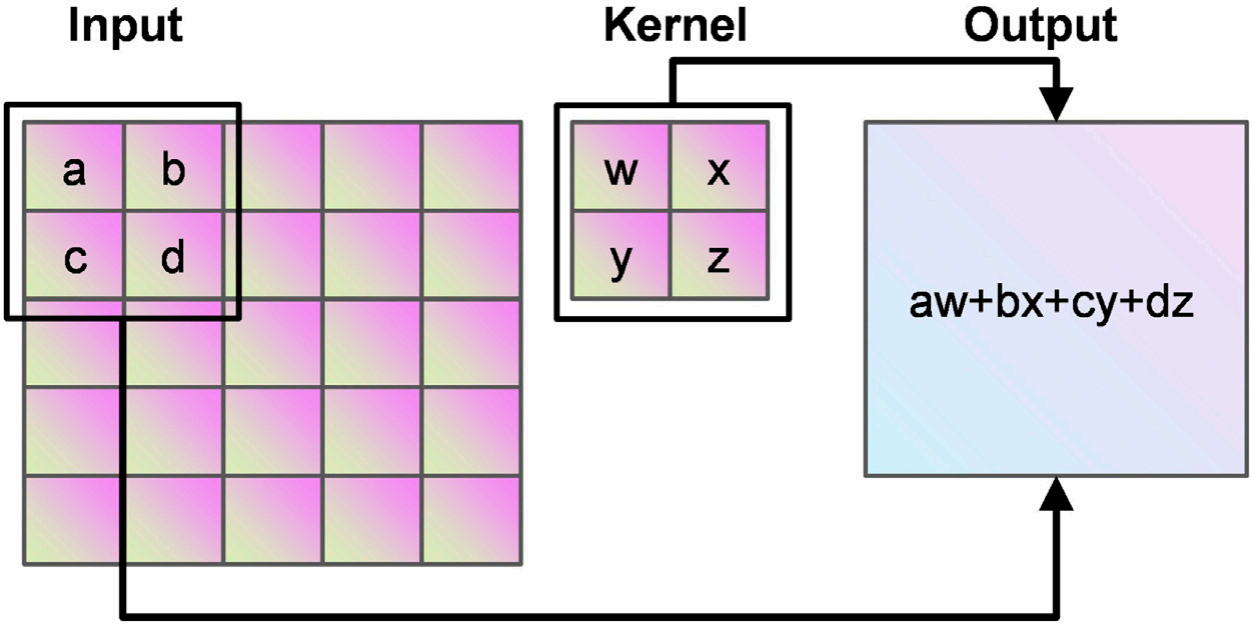
Principle of convolution.

Convolutional neural network (CNN) can learn valuable features in large-scale heart sound spectrograms developed from traditional artificial neural networks, CNN not only have the traditional fully connected neural network characteristics, but also have many differences and improvements based on them. Convolutional neural networks work on the basic principle of converting the original data into a two-dimensional matrix format, which is superior to traditional artificial neural networks in terms of the performance of extracting image feature values. In CNN, the initial convolutional layer functions similarly to an edge detector and can be used to identify low-level features. Although the network near the convolutional layer is more complex or abstract, because of the CNN weight sharing property, its network requires fewer parameters to train than the fully connected to the feature space. It shows that when the network layers, each layer output at the same time, the number of dimensions required for the stage CNN to process the same data is much lower than the whole connected to the feature space fully. Compared with other feature extraction methods, CNN has a simple structure, fitting ability and trainability. The principle of convolution calculation in CNN is shown in Figure 7.

**FIGURE 8:**
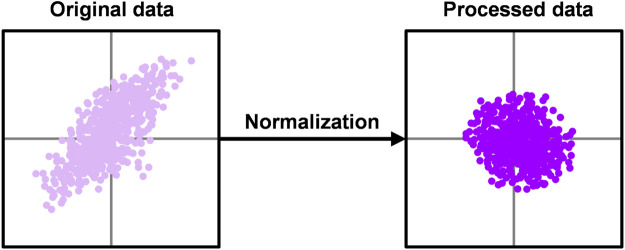
Principle of batch normalization.

Batch Normalization (BN) was originally designed to solve Internal Covariate Shift (ICS), which is a phenomenon where the internal node data distribution changes due to parameter changes in the network. ICS has a greater negative impact on deeper neural networks. Data distribution change times increase with the number of neural network layers. It makes the network harder to train and more sensitive to overfitting. BN layer adjusts their distribution by normalizing each batch of data, the principle of which is shown in Figure 8. Using the BN layer not only reduces the training time, but also make the model converge faster, and better control the problems of gradient disappearance and gradient explosion at the same time Ioffe and Szegedy (2015). The BN is calculated as follows

$${\hat{x}}_{l}=\frac{x_{i}-\mu_{B}}{\sqrt{\sigma_{B}^{2}+\epsilon }}$$ (12)

where *μ*_*B*_ is the mean of each batch of data, $\sigma_{B}^{2}$ is the variance of each batch of data, and *ϵ* is called the smoothing term, which ensures numerical stability in the operation by stopping the division by zero values.

The residual neural network was proposed initially by He et al. (2016). The degeneration phenomenon refers to the substantial decrease in model accuracy that occurs without warning as the depth of the network continues to increase. The degeneracy phenomenon makes us reflect on non-linear transformation, which significantly improves data classification. However, as the depth of the network continues to increase, we have gone too far in the non-linear transformation to achieve linear transformation surprisingly. Bottlenecks can quickly occur when training the data using CNN, and this paper introduces a residual module to address this phenomenon. It is no exaggeration to say that half of the neural networks used in computer vision today are based on Resnet and his variants.

**FIGURE 9:**
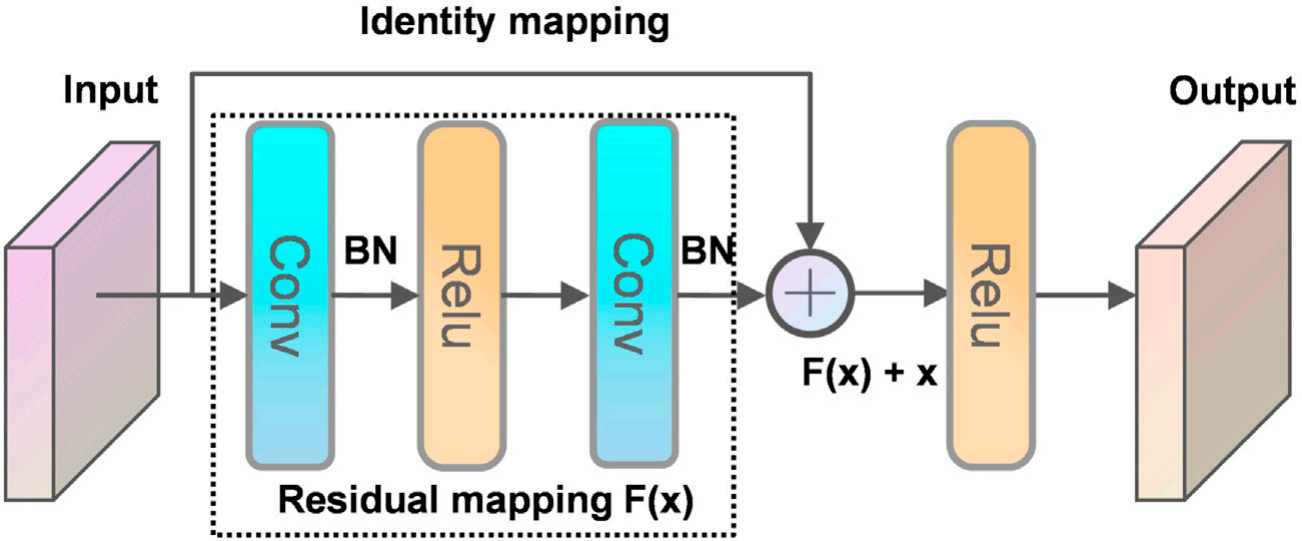
Residual structure.

The principle of the residual structure constructed in this paper is shown in Figure 9. A layer of the network can usually be viewed as y = H(x), and a residual block of the residual network is: H(x) = F(x) + x, then F(x) = H(x)—x, and y = x is the observed value and H(x) is the predicted value, so H(x)—x is the residual, that is, F(x) is the residual, so it is called the residual network. When the deep network propagates forward, the information obtained by the network decreases layer by layer as the network deepens. In contrast, ResNet deals with this problem by identity mapping. The next layer includes not only the information x of that layer, but also the new information F(x) after the non-linear transformation of that layer. This treatment makes the information instead show an increasing trend layer by layer. This is so useful that you cannot worry about lossing data. Intuitively, the residual block protects the integrity of the information by directly passing the input information around to the output, and the whole network only needs that part of the input and output difference, simplifying the experimental goal and difficulty.

## 4 Experimental evaluation

### 4.1 Dataset

This paper uses heart sound datasets published on three different platforms, the PhysioNetChallenge 2016 heart sound database, the heart sound dataset from the kaggle platform, and the Yaseen heart sound dataset. In 2016, Physionet hosted the PhysioNet/Computing in Cardiology (CinC) Challenge 2016 and released the dataset Liu et al. (2016). Physionet is a resource platform for complex physiological signal research managed by the MIT Computational Physiology Laboratory. The dataset was collected by different research groups in clinical and non-clinical conditions. These heart sound data were sampled at the same frequency, with a large amount of data and low noise. The label classification of the dataset is relatively simple and is divided into two categories: normal and abnormal. There was a wide range of audio lengths, ranging from 5 s to 120 s. In this paper, the audio was cut before the classification task. The details of this dataset are shown in Table 1.

**TABLE 1:** PhysioNet/CinC Challenge dataset.

File name	Normal	Abnormal
Training-a	292	117
Training-b	104	386
Training-c	24	7
Training-d	28	27
Training-e	183	1958
Training-f	34	80
Total	665	2575

Kaggle is currently one of the largest data science platforms in the world, with many high-quality datasets. These datasets are often sponsored by large companies for data science competitions in 2016, Kaggle held a heart sound classification competition with a dataset that referenced the Pascal heart sound dataset Jiang and Choi. (2006) and attached several description files without any modifications to the audio files. For labeling purposes, the dataset used in this paper is the one published by Kaggle. The audio lengths in this dataset range from 1s to 30 s, and the details are shown in Table 2.

**TABLE 2:** Pascal dataset.

File name	Normal	Murmur	Extrahs	Artifact
Set-a	31	34	19	40
Set-b	320	95	None	None
Total	351	133	19	40

The third dataset was open-sourced by Herzig et al. (2014) on the GitHub platform, and the authors preprocessed the dataset. The audio was sampled at the same frequency, with the same length and less murmur. The data were labeled with five categories: normal, aortic stenosis, mitral valve insufficiency, mitral stenosis, and murmur, the latter four being abnormal heart sound signals in patients with cardiovascular disease, with the specific information shown in Table 3.

**TABLE 3:** Yaseen dataset.

File name	Normal	Aortic stenosis	Mitral stenosis	Mitral regurgitation
N	200	None	None	None
AS	None	200	None	None
MS	None	None	200	None
MR	None	None	None	200
MVP	None	None	None	None

### 4.2 Experimental setup

In this study, we use Accuracy, Sensitivity, Specificity, and Precision to evaluate the proposed method. All of them are defined as follows

$$\text{ Accuracy }=\frac{TP+TN}{TP+TN+FP+FN}$$ (13)

$$\text{ Sensitivity }=\frac{TP}{TP+FN}$$ (14)

$$\text{ Specificity }=\frac{TN}{TN+FP}$$ (15)

$$\text{ Precision }=\frac{TP}{TP+FP}$$ (16)

To further illustrate the classification performance, we tested the proposed algorithm on two different deep learning network architectures by adding LSTM and GRU, whose structures are shown in Table 4. LSTM(x) represents an LSTM layer, and x is the dimension of the output space. GRU(x) represents a GRU layer, and x is the dimension of the output space. Drop(x) represents a Dropout layer, x is the possibility of dropping neurons. FC(x) represents a fully connected layer with x neurons. Conv [x, (y, z)] represents a convolution layer, x is the number of filters, y and z are the width and height of 2D filter window. BN represents a Batch Normalization layer Ioffe and Szegedy (2015). SeparableConv [x, (y, z)] is a deeply separable convolutional layer. MaxPooling (x, y) is a max pooling layer, and x and y are the pooling sizes. Residual (x) is a residual connectivity module, it is not a specific layer, it marks the position of the output layer. Add represents a residual connection layer, which takes the output of a previous layer as the input of a later one. GlobalAveragePooling() represents the global average pooling layer.

**TABLE 4:** The parameters of deep learning architecture.

Model	Structure details	Params	Training time s)
LSTM	LSTM (64)-Drop (0.5)-FC(64)-FC (3)	30,979	75
GRU	GRU (64)-Drop (0.5)-FC(64)-FC (3)	24,515	55
CNNa	Conv [16, (3,3)]-MaxPooling (3,3)-Conv [32, (3,3)]-MaxPooling (3,3)-Conv [64, (3,3)]- MaxPooling (3,3)-Conv [128, (3,3)]-MaxPooling (3,3)-Drop (0.5)-GlobalAveragePooling ()-Dense (3)	97,539	55
CNNb	Conv [16, (3,3)]-MaxPooling (3,3)-Conv [32, (3,3)]-MaxPooling (3,3)-Conv [64, (3,3)]- MaxPooling (3,3)-Conv [128, (3,3)]-MaxPooling (3,3)-Drop (0.5)-GlobalAveragePooling ()-Dense (3)	40,979	200
Resnet	Conv [8, (3,3)]-BN-Conv [8, (3,3)]-residual {Conv [16, (1,1)]-BN}-SeparableConv [16, (3,3)]-BN-MaxPooling (3,3)-add-residual {Conv [32, (1,1)]-BN}-SeparableConv [32, (3,3)]-BN-SeparableConv [32, (3,3)]-BN-MaxPooling (3,3)-add-residual {Conv [64, (1,1)]-BN}- SeparableConv [64, (3,3)]-BN-SeparableConv [64, (3,3)]-BN-MaxPooling (3,3)-add- residual {Conv [128, (1,1)]-BN}-SeparableConv [128, (3,3)]-BN-MaxPooling (3,3)-add-Conv [3, (3,3)]-GlobalAveragePooling ()	52,339	320

### 4.3 Experimental results

**FIGURE 10:**
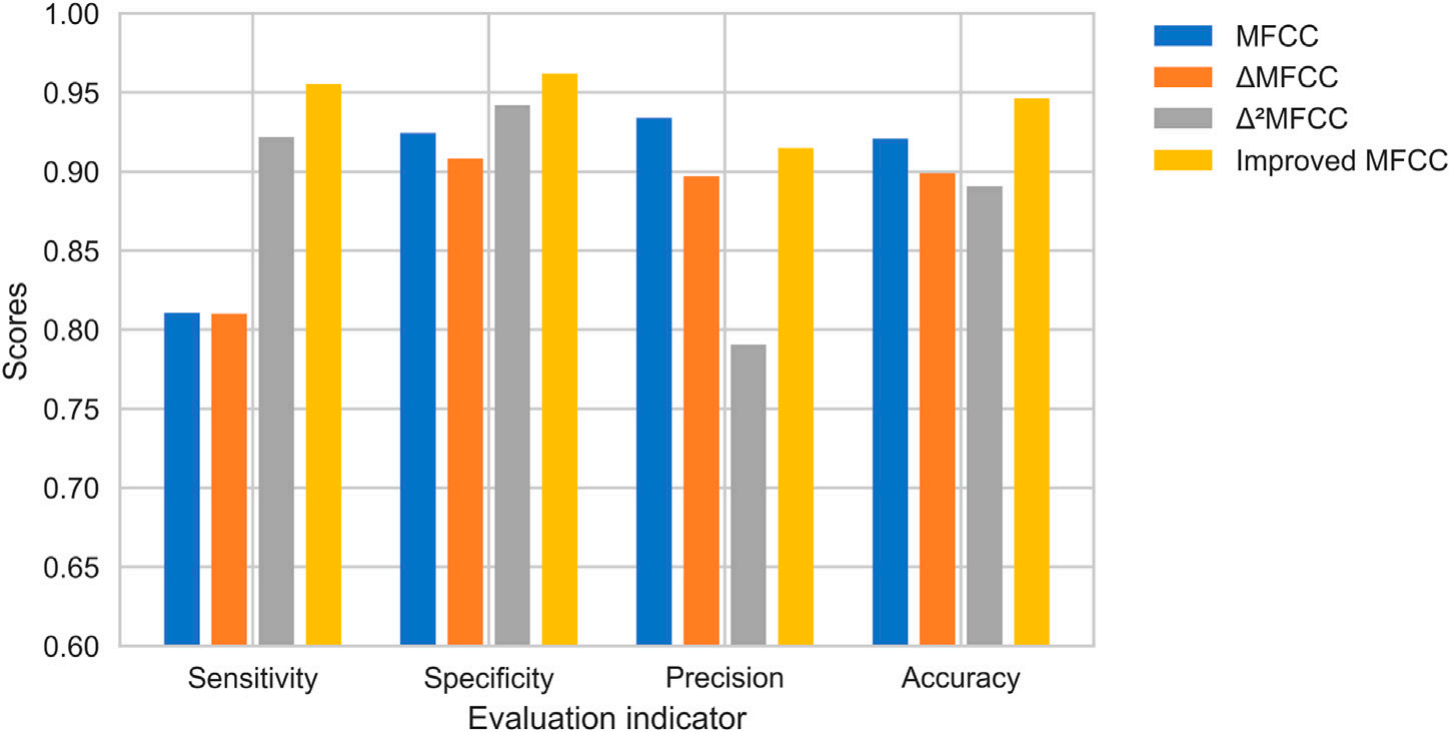
Comparison of heart sound features based on the proposed method.

To test the validation of the improved MFCC, we do comparison using the single features. MFCC, ▵MFCC, ▵^2^MFCC, and improved MFCC are trained on neural network separately, and the best epoch is taken as the result for comparison. The results of this experiment are shown in Figure 10. Improved MFCC’s sensitivity, specificity, and accuracy are higher than other features, the precision is lower than MFCC. In medical signal recognition, higher sensitivity and specificity is a valid result. Especially for sensitivity, identifying more patients is a crucial thing.

**FIGURE 11:**
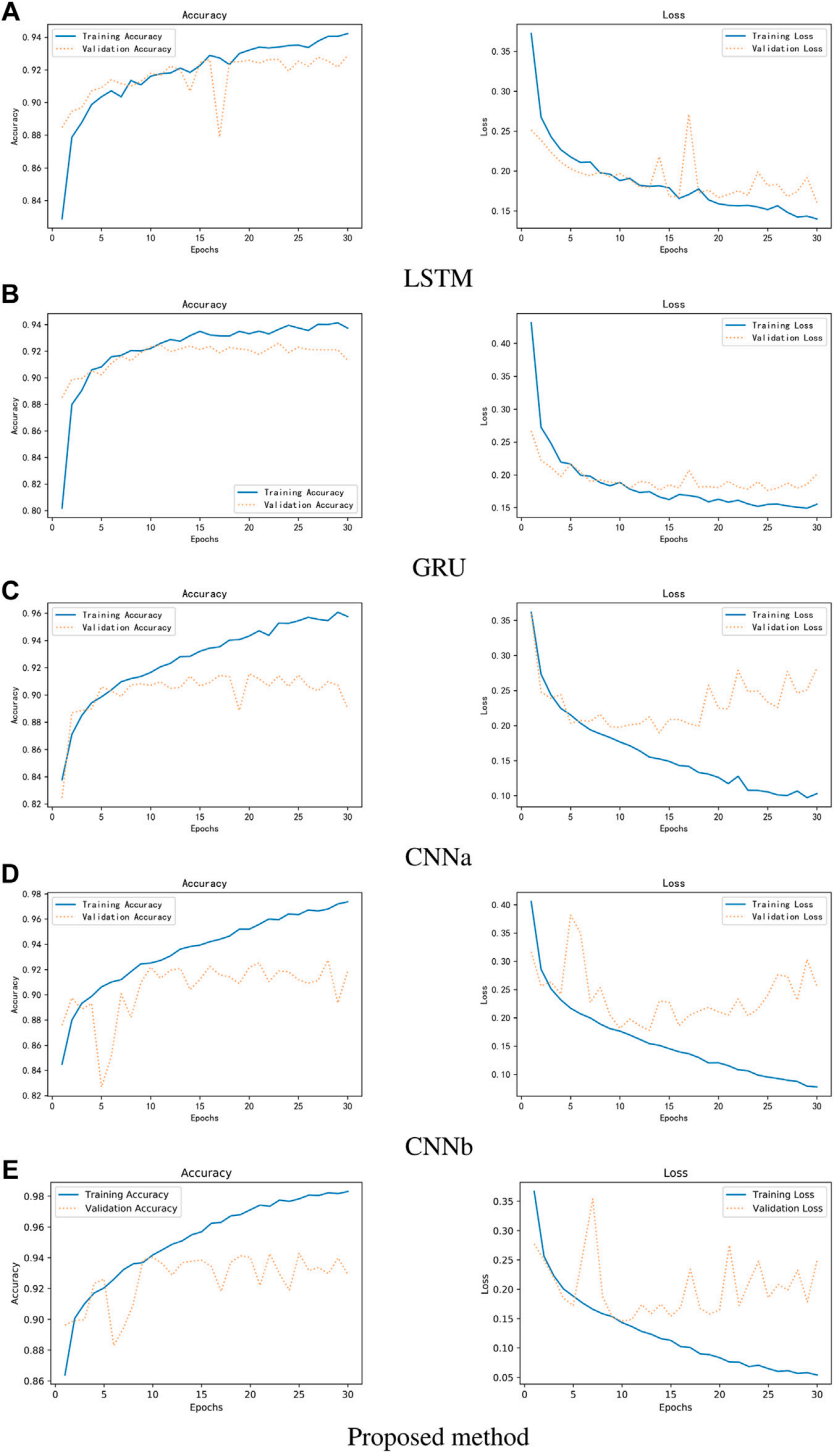
Comparison of three different networks between accuracy and loss. **(A)** LSTM **(B)** GRU **(C)** CNNa **(D)** CNNb **(E)** Proposed method.

Figure 11 shows the experimental results. It can be observed that the single Resnet, although the accuracy is higher, overfitting occurs very fast and overfitting occurs in the 10th round. Although LSTM can avoid overfitting better, has not yet reached the accuracy of Resnet in the 10th round, or even in the 30th round. This should be due to feature engineering, because the first-and second-order MFCC features are more reflective of relationships on time series, a property that is good for LSTM and GRU, but not friendly for networks like Resnet that extract locally relevant features. In addition, it can be seen that the accuracy of GRU is much lower than LSTM, but the average training time per round is 55 s for GRU and 75 s for LSTM. On the whole, Resnet can get better results.

**FIGURE 12:**
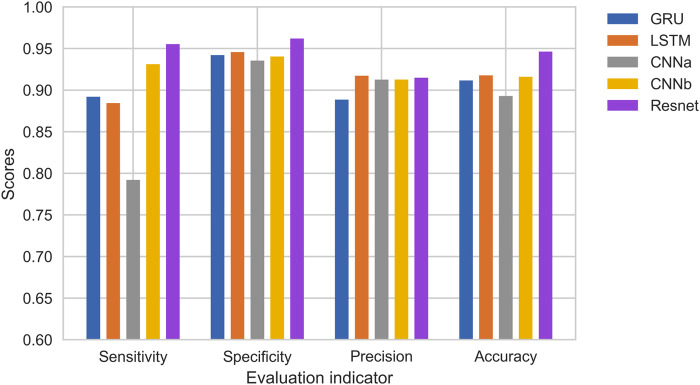
Comparison of RNNs and CNNs.

Figure 12 shows the results of the comparison. CNNa has a shallow structure. In terms of performance, it is the least effective. The CNNb structure eliminates the residual connection of the Resnet. In comparison to CNNa, it performs better. In addition, it can be seen that the accuracy of GRU is lower than LSTM. The highest score is achieved by Resnet. As a result, it was determined that deep structure and residual connections are useful for classification of heart sounds. The results shows the training process of RNNs, CNNs and Resnet. It can be observed that the CNNs and Resnet, although the accuracy is higher, overfitting occurs very fast in the 10th round. Although LSTM can avoid overfitting better, has not yet reached the accuracy of CNNb and Resnet in the 10th round, or even in the 30th round. Overfitting exists in all machine learning problems. Obtaining more authoritative heart sound data is the best solution. Adjusting the capacity of the model is another solution. For a deep learning model, the number of parameters it can learn is called the capacity. If the model has a very large capacity, then the model can even achieve a dictionary-style mapping of the data, but this mapping does not have any recognition of new data, which is a serious overfitting. So this is when we need to improve the generalization ability of the model by decreasing the capacity of the model and compelling the model to learn the most important patterns. To reduce the influence of data partitioning on the experimental results, we use 5-fold cross-validation. The first step divides 20% on the whole dataset as the test set. The second step selects 80% of the remaining as the training set and 20% of the remaining as the validation set. It will reapeat the second step 5 times to allow the validation set to iterate, each time training a new neural network separately. Finally, taking the average of the accuracy of the five models on the test set as the study result.

Table 5 shows the comparison with the results of other studies. The essential difference between CNN and Resnet is that Resnet introduces a residual structure, which effectively mitigates the effect of degeneracy on the training of deep neural networks. Thus, it can be more applicable to the heart sound classification problem. In addition to the residual structure, the features are also essential. MFCC is inspired by biology and simulates the non-linear changes of the human ear to sound, thus, extracting the physiological and pathological information in heart sounds, which can fully reflect the disease of the heart. Considering MFCC only reflects the static information of the heart sound signal, but the dynamic information of the heart sound signal also contains rich pathological features, which can be used to improve the classification accuracy further. We merge the extracted dynamic features with static features to more fully represent the physiological and pathological information in the heart sounds.

**TABLE 5:** Comparison of experimental results of different algorithms.

References	Algorithms	Sensitivity (%)	Specificity	Precision	Accuracy (%)
Li et al. (2021)	SFTF and CNN	88.70	86.40%	—	86.00
Wu et al. (2019)	MFCC and CNN	91.73	87.90%	—	89.81
Tschannen et al. (2016)	Wavelet and CNN	88.12	76.30%	—	82.12
Li F. et al. (2020)	497-features and CNN	87.00	72.10%	—	86.80
Er. (2021)	LBF and LTF	91.24	—	90.36%	91.66
Ours	Improved MFCC and Resnet	**92.32**	**95.47%**	**90.55%**	**94.43**

## 5 Conclusion

In this paper, we fused datasets from three different platforms for the lack of reliable heart sound datasets, which provided a solid foundation for neural network training. In addition, we used an enhanced feature extraction algorithm based on MFCC, and experiments show that using such features as input to the neural network can improve the model’s performance well. The proposed method makes the neural network training faster and the model generalization enhanced, which effectively mitigates the negative effects of gradient disappearance and degradation phenomena on medical signal recognition and achieves an accuracy rate of 94.43% on the constructed dataset, which is higher than the state-of-the-art methods.

## Data Availability

The datasets presented in this study can be found in online repositories. The names of the repository/repositories and accession number(s) can be found in the article/supplementary material.
